# Effect of ultrasound-guided central venous catheter insertion on the incidence of catheter-related bloodstream infections and mechanical complications

**DOI:** 10.1186/s12879-019-4487-0

**Published:** 2019-10-16

**Authors:** Osamu Imataki, Mami Shimatani, Yukiko Ohue, Makiko Uemura

**Affiliations:** 1 Division of Hematology, Department of Internal Medicine, Faculty of Medicine, Kagawa University, 1750-1 Ikenobe, Miki-town, Kita-county, Kagawa, 761-0793 Japan; 2 Nursing Division, Kagawa University Hospital, Kagawa, Japan

**Keywords:** Ultrasound-guide, Central venous catheter, Catheter-related bloodstream infection, Hematological oncology

## Abstract

**Background:**

Central venous catheters (CVCs) are necessary for critically ill patients, including those with hematological malignancies. However, CVC insertion is associated with inevitable risks for various adverse events. Whether ultrasound guidance decreases the risk of catheter-related infection remains unclear.

**Methods:**

We observed 395 consecutive CVC insertions between April 2009 and January 2013 in our hematological oncology unit. Because the routine use of ultrasound guidance upon CVC insertion was adopted based on our hospital guidelines implemented after 2012, the research period was divided into before December 2011 (early term) and after January 2012 (late term).

**Results:**

Underlying diseases included hematological malignancies and immunological disorders. In total, 235 and 160 cases were included in the early- and late term groups, respectively. The median insertion duration was 26 days (range, 2–126 days) and 18 days (range, 2–104 days) in the early- and late term groups, respectively. The internal jugular, subclavian, and femoral veins were the sites of 22.6, 40.2, and 25.7% of the insertions in the early term group and 32.3, 16.9, and 25.4% of the insertions in the late term group, respectively. The frequency of catheter-related bloodstream infection (CRBSI) was 1.98/1000 catheter days and 2.17/1000 catheter days in the early- and late term groups, respectively. In the subgroup analysis, the detected causative pathogens of CRBSI did not differ between the two term groups; gram-positive cocci, gram-positive bacilli, and gram-negative bacilli were the causative pathogens in 68.9, 11.5, and 14.8% of the cases in the early term group and in 68.2, 11.4, and 18.2% of the cases in the late term group, respectively. In the multivariate analysis to determine the risk of CRBSI, only age was detected as an independent contributing factor; the indwelling catheter duration was detected as a marginal factor. A significant reduction in mechanical complications was associated with the use of ultrasound guidance.

**Conclusions:**

Ultrasound-guided CVC insertion did not decrease the incidence of CRBSI. The only identified risk factor for CRBSI was age in our cohort. However, we found that the introduction of ultrasound-guided insertion triggered an overall change in safety management with or without the physicians’ intent.

## Background

Central venous catheters (CVCs) are a useful treatment modality for patients who require intensive critical care. A CVC allows intravenous administration of drugs and parenteral nutrition support. Although CVCs enable the delivery of medications and nutritional support that cannot be administered safely via a peripheral vein, their use is inevitably associated with adverse events, including mechanical complications, deep venous thrombosis, and catheter-related infections [1, 2]. Accordingly, the Healthcare Infection Control Practices Advisory Committee (HICPAC) guidelines for the prevention of intravascular catheter-related infections [3, 4] recommend the following procedures: hand hygiene, aseptic technique, maximal barrier precautions, skin decontamination, and catheter site dressing regimens. However, when determining additional precautions for catheter-related infections, little has been elucidated regarding whether ultrasound-guided catheter insertion reduces the incidence of such infections, including catheter-related bloodstream infection (CRBSI).

## Methods

### Study patients and inclusion criteria

We conducted a retrospective cohort study to investigate the incidence of CRBSI following CVC placement with or without ultrasound guidance. We observed 395 consecutive CVC insertions performed between April 2009 and January 2013 (3 years and 10 months). The study population included patients with hematological or immunological diseases who required CVC replacement for nutritional support and/or intravenous drug treatment.

The research period was divided into two terms based on substantial differences in the use of ultrasound-guided CVC insertion in the hematological oncology unit: before December 2011 (early term) and after January 2012 (late term). Between the early and the late terms, the insertion maneuvers changed from a blind approach to an ultrasound-guided approach after 2012.

### Treatment regimens

The following devices were used in this study: SMAC Plus MicroNeedle (15G, 13 cm or 12G, 20 cm; Covidien Tokyo Inc., Tokyo, Japan), Argyle peripherally inserted central catheter (PICC) kit (4.5Fr, 60 cm; Covidien Tokyo, Japan), Arrow triple lumen (7Fr; Arrow, Tokyo, Japan), and Blood Access UK catheter kit, double (11Fr; UNITIKA, Tokyo, Japan).

All of the practitioners involved in this study had attended a hospital training program providing information on the standard insertion technique. The need for a CVC was determined by each practitioner; the practitioners also determined the preferred CVC device and the insertion site for each patient. Maximal sterile barrier precautions were routinely adopted. A 10% tincture of povidone iodine was used for skin preparation, and Tegaderm Transparent Film Dressing (3 M Japan, Tokyo, Japan) was used as the routine catheter site dressing unless otherwise specified. Study competency in CVC insertion was guaranteed by the training of the CVC providers. Our institute demands institutional certification to perform CVC/PICC insertion, which is only granted after the regulated orientation and training program and three instances of practical performance under a qualified instructor. All the CVC/PICC providers, who were a combination of staff and residents, were certified according to Kagawa University Hospital institutional regulation. The staff practitioners (*N* = 24) all had > 4 years of experience (median 5, range 4–15 years) in CVC/PICC intervention performance. The residents (*N* = 32) had < 4 years of experience (median 2, range 1–3 years). We used 10% povidone iodine solution for skin decontamination for the preparation of CVC/PICC insertion. Chlorhexidine gluconate dressing (CHGD) was applied as a form of infection prevention for stem cell transplantation. Levofloxacin and azoles were used as prophylactic antimicrobials during chemotherapy. Other interventions were not applied.

### Definition

A bloodstream infection (BSI) [5] was defined by the first set of positive blood cultures in a series [6]. To distinguish between a true BSI and contamination, more than two investigators critically analyzed the blood culture results. In addition, a CRBSI [5, 7] was defined as a positive culture result from at least one peripheral blood sample, a catheter tip culture positive for the same microorganism as the peripheral blood sample, and clinical signs of bacteremia. The concomitance of any other source of bacteremia was clinically assessed. The diagnosis of catheter-related infection was made in accordance with the HICPAC guidelines definition [4]. Mechanical complications included arterial puncture, hematoma, and pneumothorax. The grading scales used in assessing complications were from the Common Terminology Criteria for Adverse Events Version 4.0 https://ctep.cancer.gov/protocolDevelopment/electronic_applications/CTC.htm. The grade 1 to 4 pneumothoraxes are asymptomatic, symptomatic, sclerosis and/or operative intervention indicated, and life-threatening, respectively. The grade 1 hematoma has mild symptoms, grade 2 has minimally invasive evacuation or aspiration indication, grade 3 has transfusion or radiologic, endoscopic, or elective operative intervention indicated, and grade 4 has life-threatening consequences. The grade 1 bleeding has mild symptoms, grade 2 has medical intervention indicated, grade 3 has transfusion or radiologic, endoscopic, or operative intervention indicated, and grade 4 has life-threatening respiratory or hemodynamic compromise. We evaluated comorbidities using the Charlson comorbidity index, which is a common and useful tool for evaluating general complications, and organ dysfunction [8]. The primary endpoint of the study was to determine the effect of ultrasound guidance in CVC insertion on the incidence of CRBSI.

### Statistical analysis

We used basic statistics and described representative values for the patients’ backgrounds. To compare values between the two groups, a two-tailed paired Student’s *t*-test was used for the parametric analyzes, and the Wilcoxon signed-rank test was used for the non-parametric analyzes. The contributing risk factors for catheter-related infection were analyzed using the multivariate analysis method. For the multivariate analysis, we evaluated patient background (age, sex, and clinical characteristics) and catheter conditions (insertion site, catheter indwelling duration, catheter insertion situation, and catheter device) as independent variables, and the onset of CRBSI as a dependent variable using the regression model. All potential confounders were included as independent variables. A stepwise selection procedure was used to build the multivariable logistic regression model using the above background risk variables. The entry criterion was set at *P* < 0.15. Statistical significance was defined as *P* < 0.05. An interrupted time-series analysis (TSA) was performed to analyze the trends and test the significance over the study period [9]. The *t*-test or Chi-squared test was used to compare values between the two groups. For the comparison tests, *P*-values < 0.05 were considered significant. Statistical analyzes were performed using SPSS version 19.0 J software (SPSS Japan, Tokyo, Japan).

### Ethical issues

This study was conducted in accordance with the ethical standards of the responsible committee on human experimentation (Kagawa University Hospital Institutional Review Board, IRB) and with the Declaration of Helsinki (1964, amended most recently in 2008) of the World Medical Association.

## Results

**Fig. 1:**
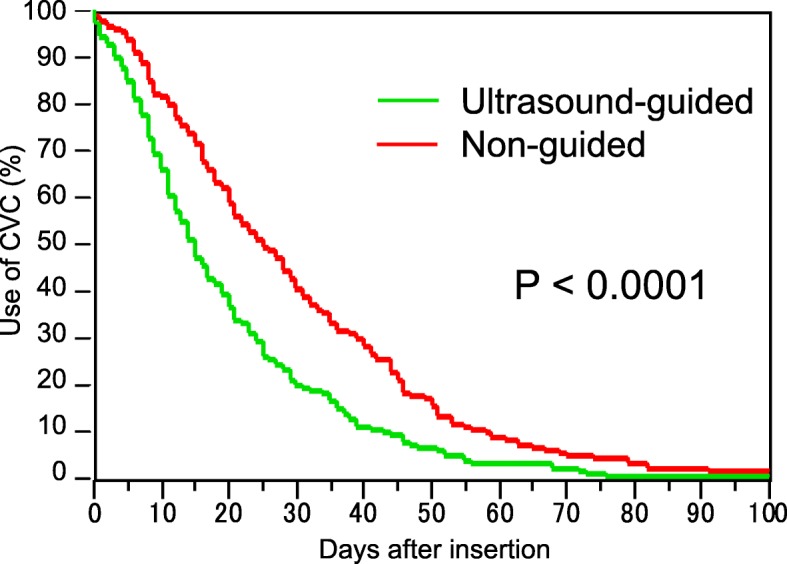
Insertion duration. The insertion duration was significantly shorter in the late term than in the early term (*P* < 0.0001)

The patients’ characteristics are presented in Table 1 and Fig. 1. A total of 395 insertion cases were surveyed in the hematological oncology unit. The underlying conditions of the patients included hematological or immunological diseases, including hematological malignancies (*n* = 340), immunological disorders such as autoimmune diseases (*n* = 35), and solid malignancies (*n* = 20).

**Table 1:** Patients’ characteristics according to the study period

		Early term Non-guided	Late term Ultrasound-guided	*P*-value
Number of patients		235	160	–
Median age (range)*		59 (19–85)	53 (20–78)	< 0.0001
Gender (female/male)*		122/133	101/59	0.0018
Insertion duration*, median (range) [days]		26 (2–126)	18 (2–104)	< 0.0001
Charlson comorbidity index		2.9 (0–11)	2.8 (0–8)	0.681
Hospitalization days		90 (1–318)	66 (3–308)	0.0002
Total observation [catheter days]		7210	3231	–
Site direction (%)	Right	76.3	78.1	0.599
	Left	17.5	14.6	
Insertion site (%)	Internal jugular	22.8	32.5	< 0.0001
	Subclavian*	44.7	19.9	
	Femoral	31.1	33.8	
	Others	0.4	13.2	
Number of lumens (%)	Single	2.2	9.3	0.0348
	Double*	86.4	68.2	
	Triple	8.3	15.2	
	Others	1.3	0.7	
Usage of PICC*, among all cases (%)		0.4	13.2	0.0003

*P < 0.05

**Fig. 2:**
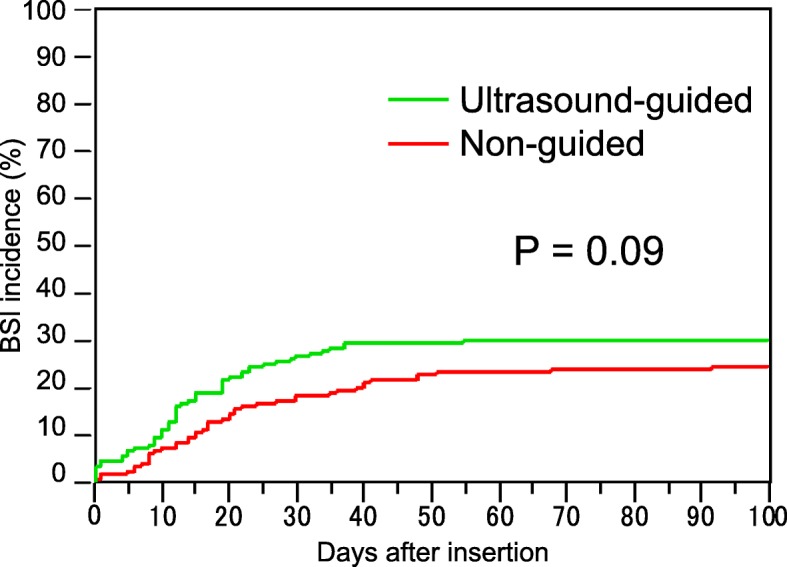
Cumulative incidence of CRBSI. The cumulative incidences of CRBSI at 100 days in the early and late terms were 25.4 and 30.8%, respectively. Although the accumulation was slightly higher in the late term than in the early term, the difference was not significant (*P* = 0.09)

**Fig. 3:**
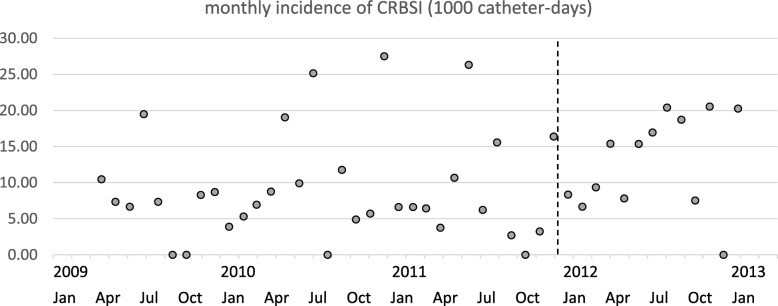
Monthly time-series incidence of CRBSI. The interrupted time-series analysis did not reveal a significant gap and trend of the impact of the ultrasound-guided insertion maneuver according to the time-varied incidence before and after the intervention (dotted line, between December 2011 and January 2012)

Of the 395 cases recruited in this study, 235 cases were included in the early term and 160 in the late term. The median insertion duration was 26 days (range, 2–126 days) in the early term and 18 days (range, 2–104 days) in the late term (Table 1). The insertion duration was significantly shorter in the late term than in the early term (*P* < 0.0001; Fig. 1). We observed 7210 and 3231 catheter days in the early and late terms, respectively (Table 1). During the early term, 22.8, 44.7, and 31.1% of the insertions were performed at the internal jugular, subclavian, and femoral veins, respectively. During the late term, 32.5, 19.9, and 33.8% of the insertions were performed in these respective places (Table 1). The clinical outcomes are summarized in Fig. 2. The ultrasound-guided insertion approach became a routine practice in the late term. The frequency of CRBSI was 1.98/1000 catheter days and 2.17/1000 catheter days in the early and late terms, respectively. The cumulative incidence of CRBSI at 100 days was 25.4 and 30.8% in the early and late terms, respectively (Fig. 2). The accumulation was slightly higher in the late term than in the early term; however, the difference was not significant (*P* = 0.09; Fig. 2). The post-hoc estimated power was 0.177. We also performed an interrupted TSA with a Poisson regression model using monthly rates of CRBSIs. This analysis did not reveal a significant difference between before and after the ultrasound-guided method. This analysis supported the counterfactual “the incidence trend of CRBSI continues unchanged pre- and post-intervention period” (Fig. 3). The average incidence of CRBSI was 9.15 (standard deviation, 7.41) and 12.87 (6.62) in the pre- and post-intervention periods, respectively (*P* = 0.1215). The observed effect size *d* was calculated as 0.530, which was an intermediate effect size.

Mechanical comorbidities decreased from 0.055 (13/235) to 0.000 (0/160) incidences/insertions after the introduction of the ultrasound-guided insertion approach. The cumulative incidence of mechanical comorbidities was 2.01 and 0.00% per insertion, respectively. The observed complications were grade 2 pneumothorax in two cases, grade 3 pneumothorax in eight cases, and grade 2 hematoma with grade 1 bleeding in three cases. In the subgroup analysis, the proportion of detected causative pathogens in the CRBSI cases did not differ between the two terms; gram-positive cocci, gram-positive bacilli, and gram-negative bacilli were the causative pathogens in 68.9, 11.5, and 14.8% of the cases, respectively, in the early term and in 68.2, 11.4, and 18.2% of the cases, respectively, in the late term. In the multivariate analysis to determine the risk of CRBSI, only age was detected as an independent contributing factor (*P* = 0.0091); the indwelling duration was detected as a marginal factor (*P* = 0.0868) (Table 2).

**Table 2:** Multivariate analysis for the contributing factors of CRBSI

Clinical situations	Contributing factors	Odds ratio	95% CI	*P*-value
Patients’ characteristics	Age	3.520	0.003~0.032	0.0091*
	Sex	1.380	−0.078~0.407	0.2270
	Absolute neutrophil upon insertion	−0.275	−0.001~0.001	0.4557
Catheter conditions	Resident performance	0.807	−0.742~0.328	0.2257
	Insertion direction (right or left)	1.064	−0.282~0.329	0.6399
	Insertion site (internal jugular, subclavian, or femoral)	0.987	−0.424~0.415	0.6888
	Catheter indwelling duration	0.013	−0.023~0.002	0.0868
	Ultrasound-guided	0.833	−0.635~0.274	0.7201
	Number of lumens	0.787	−0.021~0.001	0.9475
	Size of catheter	1.365	−0.085~0.398	0.4877

*CI* confidence intervals

*P < 0.05

The controlled-cohort study revealed that CHGD use decreased the incidence of CRBSI caused by *Staphylococcus* species (1/11 cases versus 11/34 cases with and without the use of CHGD, respectively), but this difference was not significant (*P* = 0.1294). The overall incidence of bacteria including other organisms was comparable in the two groups at approximately 30% each (3/11 cases versus 16/34 cases, respectively; *P* = 0.2481). In cases without CHGD use, the documented organisms included *Staphylococcus epidermidis* (*n* = 6), methicillin-resistant*S. aureus* (*n* = 1), *S. hominis* (*n* = 2), *S. simulans* (*n* = 1), *Staphylococcus* species (*n* = 1), *Streptococcus* species (*n* = 1), *Capnocytophaga* species (*n* = 1), *Stenotrophomonas maltophilia* (*n* = 1), *Escherichia coli* (*n* = 1), and gram-negative bacilli (*n* = 1) (unidentified species). In cases with CHGD use, *S. epidermidis* (*n* = 1), *Stenotrophomonas maltophili*a (*n* = 1), and *Enterococcus faecium* (*n* = 1) were documented.

## Discussion

Ultrasound-guided CVC insertion did not significantly decrease the incidence of CRBSI in this study. A previous study reported the incidence of CRBSI to be 24.5% in hematological malignancies, including in recipients undergoing stem-cell transplantation [10], which is comparable to the rate observed in our study. The interrupted TSA also did not find clear differences between before and after ultrasound-guided insertion was applied. This analysis was adjusted for time-varying cofactors, such as line type, insertion site, and duration of line use. The other concomitant differences observed after the introduction of the ultrasound-guided approach in our cohort survey can be summarized as follows: (1) a significant decrease in insertion using the subclavian vein approach, (2) a clear increase in insertion using the internal jugular vein approach, (3) a significantly shorter CVC insertion duration, (4) a preference for the right site direction, (5) reduced double-lumen catheter use, and (6) positive selection of the PICC insert. All of these were intentional trends in our hospital related to a decrease in CVC-related complications. These interventions were partially recommended by the Department of Safety Management in our hospital. Although all of these changes may contribute to a decreased incidence of BSI, the patient-day-based incidences of BSI and CRBSI did not decrease during the study period. In contrast, mechanical complications significantly decreased after the introduction of the ultrasound-guided CVC insertion approach. The trend of CVC insertion maneuvers for improving patient safety reported in our study led to in-house preference recommendations: (1) a shorter insertion period and early catheter removal, (2) a right internal jugular vein approach using a single lumen, and, if applicable, (3) the use of PICC inserted under ultrasound-guided puncture with CHGD dressing. We believe that these recommendations are required to prevent multiple adverse risks including BSI. We advocate that the trend of CVC insertion maneuvers should be based on the governance for safety among community opinions.

The ultrasound-guided CVC cannulation is a safer procedure than the landmark technique [11]. Previous studies have suggested that ultrasound-guided catheter insertion results in other favorable benefits as well, such as an increased rate of successful catheterization [12]. In one study, authors reported a 100% success rate with ultrasound guidance [13]. Furthermore, a valuable movie indicating a detailed technique and methodology can be previewed in an electronic journal [14]. Additional reported benefits include lower mean access time and mean attempt number [13] and reduced complications, such as hematoma, carotid puncture, hemothorax, and pneumothorax [13, 15, 16]. However, the authors in that report did not evaluate other late incidental complications, including local site infection, deep venous thrombosis, CRBSI, and catheter occlusion. Our study cohort also indicated a striking reduction of mechanical complications—pneumothorax, hematoma, and thoracic bleeding. With the PICC approach, it is anatomically impossible to puncture in error. But ultrasound-guided cannulation comprehensively achieved an extremely high success rate, 98.32%, in a recent report [15]. Ultrasound-guided cannulation can be adopted at any site—internal jugular vein, subclavian vein, brachiocephalic vein, and so on—and is not limited to adult or child patients [12–15, 17–19]. The wide acceptance of ultrasound-guided insertion makes it a standard procedure for CVC/PICC [17, 20]. This safety is also achievable by the environmental factors [21].

Our survey demonstrated an intervention that triggered an overall shift in the management of CVC insertion to minimize the risks contributing to mechanical complications (pneumothorax and hematoma or other organ injuries) [18], which is a key CVC-related adverse event, whether it is intentional or unintentional. Training time is needed to increase the skill in the use of the ultrasound technique by any providers [22, 23]. But we did not observe a time lag in the effect of ultrasound guidance [23]. The ultrasound technique brought a quick and significant improvement in the safety of CVC insertion [23]. We did not evaluate other CVC-related adverse events, such as catheter-related thrombosis or embolism, because these events are not directly associated with ultrasound-guided insertion. Nevertheless, the incidence of CRBSI did not change after the introduction of ultrasound-guided CVC insertion. A major limitation of our study was its non-randomized, observational cohort design. Another central limitation of our study was the large differences in the patients’ background characteristics. The complete exclusion of bias should be qualified and warranted by further prospective randomized studies. Another limitation of our investigation is the possibility of misclassification bias of the CRBSI diagnosis because the quantitative catheter tip culture was not the standard clinical practice in our institute throughout the study period. Finally, ultrasound guidance should only be performed by practitioners fully trained in this technique. After all, a new controlled and randomized study in the future to assess the efficacy of ultrasound guided CVC insertion in CRBSI rates would be warranted.

## Conclusion

We found no definitive evidence of reduction in the incidence of CRBSI following ultrasound-guided CVC insertion. However, we found that the introduction of ultrasound-guided insertion triggered an overall change in safety management with or without the physicians’ intent.

## Data Availability

The datasets used and analyzed during the current study are available from the corresponding author on reasonable request.

## Abbreviations

BSI: Bloodstream infection
CHGD: Chlorhexidine gluconate dressing
CRBSI: Catheter-related bloodstream infection
CVC: Central venous catheter
HICPAC: Healthcare Infection Control Practices Advisory
IRB: Institutional review board
TSA: Interrupted time-series analysis
